# Antimicrobial Resistance Situational Analysis 2019–2020: Design and Performance for Human Health Surveillance in Uganda

**DOI:** 10.3390/tropicalmed6040178

**Published:** 2021-09-29

**Authors:** Ibrahimm Mugerwa, Susan N. Nabadda, Janet Midega, Consolata Guma, Simeon Kalyesubula, Adrian Muwonge

**Affiliations:** 1 National Health Laboratories and Diagnostic Services, Antimicrobial Resistance National Coordination Centre (AMR-NCC) for Human Health, Ministry of Health, Butabika, Kampala 10312, Uganda or sndidde@yahoo.co.uk (S.N.N.); or myconso@yahoo.com (C.G.); simeonk959@gmail.com (S.K.); 2 The Welcome Trust, 215 Euston, London NW1 2BE, UK; J.Midega@wellcome.ac.uk; 3 Division of Genetics and Genomics, Roslin Institute, College of Medicine and Veterinary Studies, The University of Edinburgh, Edinburgh EH25 9RG, UK; adrian.muwonge@roslin.ed.ac.uk

**Keywords:** antimicrobial resistance (AMR), sentinel surveillance, one health approach, national action plan (NAP)

## Abstract

Antibiotic resistance and its mechanisms have been known for over six decades, but global efforts to characterize its routine drivers have only gained momentum in the recent past. Drivers of clinical and community resistance go beyond just clinical practice, which is why one-health approaches offer the most realistic option for controlling antibiotic resistance. It is noteworthy that the emergence of resistance occurs naturally in the environment, but akin to climate change, the current accelerated emergence and spread bears hallmarks of anthropomorphic influence. If left unchecked, this can undo the medical and agricultural advancements of the last century. The WHO recommends that nations develop, adopt, and implement strategies that track the changing trends in antibiotic resistance levels to tackle this problem. This article examines efforts and progress in developing and implementing a human health antimicrobial resistance surveillance strategy in Uganda. We do so within the context of the National Action Plan for tackling antimicrobial resistance (AMR-NAP) launched in 2018. We discuss the technical milestones and progress in implementing surveillance of GLASS priority pathogens under this framework. The preliminary output of the framework examines the performance and compares AMR and AMU surveillance data to explain observed trends. We conclude that Uganda is making progress in developing and implementing a functional AMR surveillance strategy for human health.

## 1. Introduction

Globally, bacterial infections cause acute and chronic life-threatening infections and become difficult to treat due to the increasing emergence and spread of antimicrobial resistance. We have known about resistance and its underlying mechanism for close to seven decades, according to [1], but efforts to tackle resistance in routine practice are only starting to gain momentum. The drivers and determinants of antimicrobial resistance are complex and cut across strata of life and ecosystems, i.e., microbial, niche, food systems, human health and the environment in general [2]. According to Jim O’Neill’s report, the economic burden of AMR is global and will lead to health and social-economic challenges, especially in developing countries https://apo.org.au/node/63983 (accessed on 18 September 2021). Indeed, antimicrobial resistance is a natural phenomenon; however, its rapidly increasing prevalence is driven by misuse and overuse of antibiotics [3,4]. If not addressed, antimicrobial resistance will cost the global economy over 100 trillion USD by 2050, with a projection of over 10 million death annually [5]. To this end, AMR surveillance is considered a high-yield investment that countries must undertake to mitigate this global threat. AMR surveillance is defined as an ongoing systematic collection of samples to isolate sentinel organisms, examine their antibiotic susceptibility status, to use the output to guide planning, implementation and evaluation of national action plans. Through surveillance, countries can monitor trends and detect the magnitude of the emerging resistance to priority antibiotics. Most of all, inform national and sub-national level in areas of treatment guidelines and infection prevention and control strategies. 

Through its global Antimicrobial Resistance Surveillance System (GLASS), the World Health Organisation provides baseline guidelines for surveillance, and most national action plans share the ambitions set out in this document. The guidelines serve as a framework to safeguard the integrity of antimicrobials whose utility is critical for infection management. For example, Uganda’s national action plan (NAP) aims to prevent, slow down and control the spread of resistant pathogens, as indicated on http://www.cphl.go.ug/policy-documents (accessed on 18 August 2021). A robust surveillance system is integral to achieving these aims. Since its publication in 2018, various stakeholders along the AMR chain (human, animal health and environment) have been involved in developing and implementing components of the NAP. Documenting the progress and challenges of AMR control activities is the focus of this article. By doing so, we not only maintain the momentum to tackle AMR [3] but provide estimates for developing countries, which are more than likely to be dis-proportionately affected if this momentum subsides [3]. The fragility of the health care systems and, therefore, ease of access to antimicrobials, has pro-moted self-medication and inherently shaped the epidemiology of AMR in these countries for years. 

These antibiotics, humanity’s great weapon against infectious diseases, are becoming less effective at killing bacteria, and thus eroding their utility, especially in tropical settings. Therefore understanding the trends of resistance and their drivers allows us to develop targeted awareness campaigns especially linked to the rational use of antibiotics. 

In low and middle income countries it isn’t easy to promote rational use if health care systems are too weak to provide the necessary safety-net. For example; In Ethiopia, the problems have been highlighted from case finding, triaging, to managing infectious diseases. Poor outcomes represent risks, especially among parturients attending maternity [6].

Here, we focus on AMR surveillance in human health, mainly how systems and structures are set up and managed to avoid duplication of resource allocation and preliminary output between October 2018 and September 2019. Examining the output from this surveillance system allows us to determine (a) its utility, (b) performance and (c) challenges as it is being developed and implemented within the NAP.

### 1.1. Design of Sentinel Sites

#### 1.1.1. Establishment of AMR Governance Structures at National and Sub-National Level

**Figure 1:**
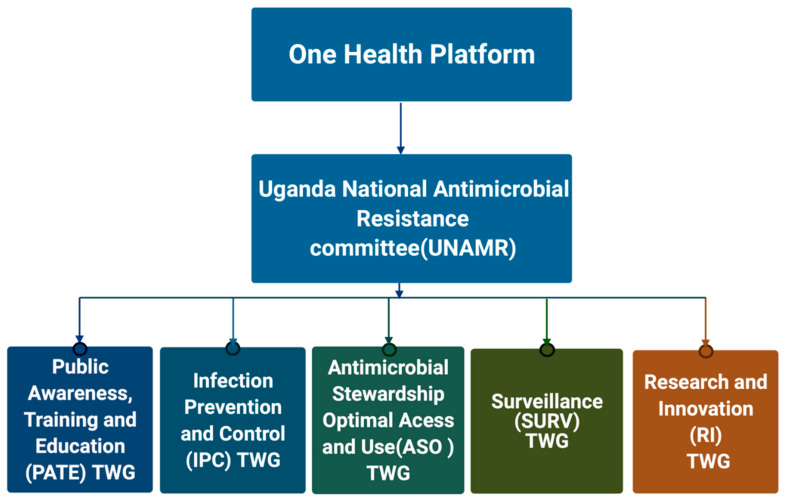
The design of Uganda’s AMR governance structure at national level. On top is the One health platform, under which is the committee that oversees activities on AMR in Uganda. The oversee the pillars on which the National action plan is implemented todate.

Governance structures are fundamental and pertinent for the pragmatic implementation of any strategy. Uganda’s AMR surveillance system has been rooted and structured in a governance structure that embraces One Health. This structure comprises the One Health Platform, the Uganda National AMR Committee and the different AMR Technical Working Groups defined by the National AMR Action Plan (Figure 1). These as well address the implementation component for the key strategic objectives of the AMR-NAP-2018.

**Figure 2:**
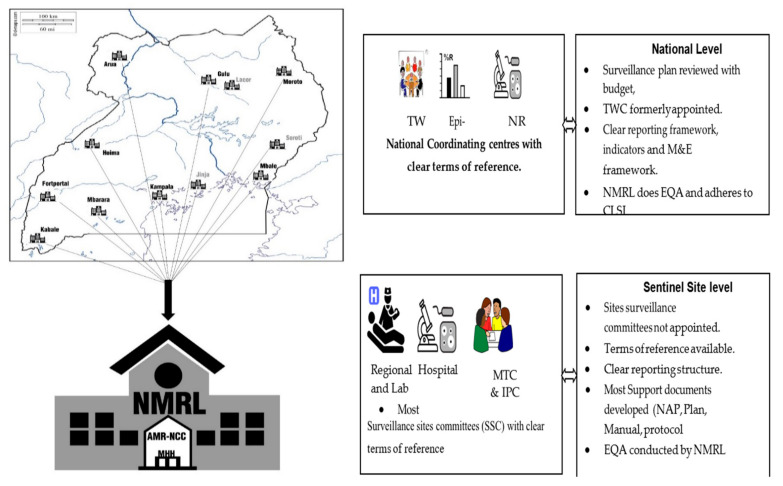
Uganda’s design of AMR Sentinel Surveillance system based on the already-existing Ministry of Health structure of regional referral hospitals and how functional linkages for conducting AMR surveillance from the facility level are extended to the national level.

This situational analysis was guided by the following central documents on AMR at the global and local levels; (a) WHO guideline on AMR published in [7], particularly the recommendations to establish national coordination centres for in-country AMR activities. The centres then become the regional or indeed sub-regional anchors of surveillance systems. In Uganda, these sites are distributed across the country and its regions and serve as sentinel sites for surveillance (Figure 2). Uganda’s AMR-NAP is a blueprint for the strategic plan on how the country will tackle AMR, and it recommends the development of critical documents to implement surveillance. The following documents were developed in the first year of the AMR-NAP; (a) Human Health AMR Surveil-lance Plan, (b) Human Health Antimicrobial Use and Consumption Surveillance Plan, (c) Diagnostic Stewardship Manual for AMR Site Surveillance and Clinical Protocols, (d) In-service Microbiology Training Curriculum, the Uganda National Policy for Bio-banking and Achieving Microbiology Isolates, and twelve sets of standard operating procedures (SOPs), which can be harmonized and adopted by all microbiology laboratories in the network with the inclusion of animal health, environment, and food and beverage laboratories. Taken together, these documents define the standard operating procedures for the delivery of the various components of the surveillance plans under the following key pillars.

The design is based on several regional referral hospitals and some high-volume private facilities. These share antimicrobial susceptibility data and isolates with the National Microbiology Reference Laboratory (NMRL). All of this surveillance and system design work is coordinated by the National AMR Coordination Centre, which is housed within the Ministry of Health Department of Laboratory (NHLDS).

#### 1.1.2. Human Health AMR Laboratory Diagnostics

Sample collection is designed to reflect the referral system of the healthcare system in Uganda, allowing for examining the changes in AMR profiles as patients are escalated through the referral system. Since patients spend long periods in hospitals at higher strata of the referral system, we expect the odds of exposure to hospital-generated AMR to increase, and the detectable resistance will increase [8]. The location of sentinel sites is critical as it directly impacts the time of sample collection to processing. This in turn has an effect on the recovery of microbes for AMR susceptibility profiling in this analysis. Of the cultured samples, 398 recovered 45 unique microbes, as shown in Figure 2. This represents a 33% culture recovery, and these included some of the GLASS priority organisms. This culture recovery rate is lower than the isolates per site annually, as set by the site performance indicators (Figure S1) target level depending on the specimen. This likely reflects the challenges in the chain between sample collection and processing. It could also reflect inefficiencies in quality assurance in the diagnostic laboratories but highlights areas for monitoring and improvement in this surveillance framework.

The recovered bacteria predominately include *E. coli*, *S. aureus*, *K. pnuemoniae*, *C. freudii* and *C. albicans* originating from urine, sputum, pus and urethral, vaginal and cervical swabs. A majority of these microbes were linked to samples collected at the Kabale sentinel site. Here we note that Mbarara recovered comparatively fewer organ-isms. The reasons are worth investigating, but it is worth noting that this is preliminary output. The GLASS sentinel organisms were recovered at all sentinel sites, reflecting their priority status as part of this surveillance. Notably, *N. gonorrhea* and *Salmonella* species were the microbes least recovered, likely a reflection of the prevalence of these infections at the sentinel sites.

**Figure 3:**
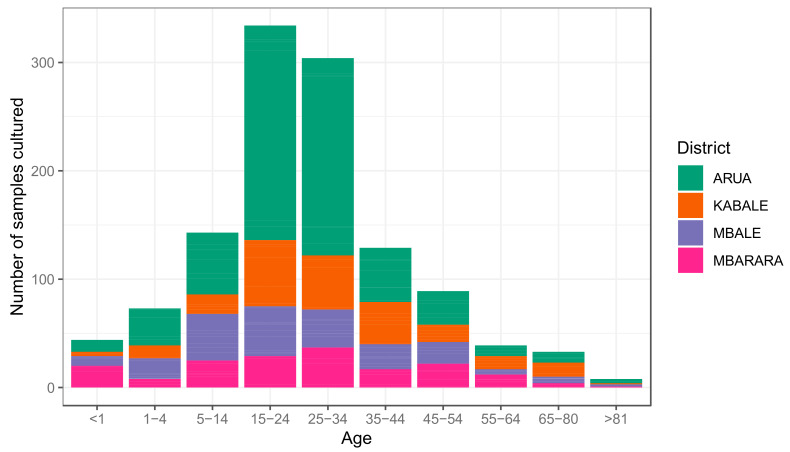
Distribution of the 1209 samples cultured from the four sentinel sites whose results are presented in this report. The *X*-axis represents the age brackets of the patients from whom samples were collected. The *Y*-axis shows the number of samples.

Figure 3 shows that most of the recovered samples were collected from female patients; here, it is evident that many of the recovered samples originated from Kabale district despite most samples originating from Arua. Most of the recovered samples were from urine and urogenital specimens, likely reflecting the clinical presentation in the 15−34-year-old patients in these sentinel districts. According to [9], it is evidence that women aged 15 to 35 years are regarded as sexually active and thus have higher exposure to urogenital infections. This could reflect the high prevalence of urogenital infections in this age group in the Kabale district. Blood appears to be the second-largest source of recovered microbes among the 1−14-year-old patients; this reflects the predominance of bacteraemia among infants in most districts.

#### 1.1.3. Integration and Development of Linkages for Electronic Data Interoperability and Sharing

Uganda has leveraged existing Ministry of Health laboratory information platforms built on the health laboratory infrastructure to form a laboratory information system known as the African Laboratory Information System (ALIS). This information system has been developed as a generic tool for the country’s laboratory system and programmed to integrate with other systems and tools like WHO-NET. The system takes all the critical variables on the patient laboratory request form (Table S2) to automatically analyse patient-level data to generate epidemiological reports. The system in development and piloted at the National Reference Laboratory can interoperate with different micro-biology equipment platforms and technologies to share data in the form of analytical reports with the National AMR Coordination centre. We have piloted this electronic laboratory information system in at least four AMR Sentinel sites. These results are reported in this document and form the foundation for our AMR surveillance. Finally, our strategy is to develop an AMR real-time surveillance dashboard to inform local facility decision-making and resource allocation at the sub-regional and national levels. It is this information that would be shared with WHO via WHO-NET for the global AMR reports.

#### 1.1.4. AMR Data Management at Facility and Reference Laboratory Level{ TC “AMR Data Management at Facility and Reference Laboratory Level” \f C \l “1” }

At the sub-national facility level, in the microbiology laboratory, request forms and the daily activity registers (Table S2) patient-level data is entered into WHONET. This software application analyses these data, and working with the facility implementing partner, data entry is validated at the facility level before being shared with the National Coordination Centre. Data validation at the national level is performed by the Technical Working Committee on surveillance that scrutinises the data before external uploading it onto the WHO-GLASS. SOPs and developed guidelines for data collection, sampling and reporting in surveillance sites are standardised: strengthen data management at AMR secretariat, NCC and sentinel sites by delivering IT infrastructure, paper-based forms and registers to sites, e.g., microbiology referral forms and registers; and train laboratorians to analyse microbiology lab AST data. Anonymised isolate level data are submitted to the AMR-NCC through an electronic system at the national level. This will eventually link with the national database (DISC) to evaluate the quality and report monthly AMR and microbiology data to DHIS2.

At the national level, patient-level data are received with their parent isolates from their respective facilities. They are then anonymised for analysis using WHONET software that is integrated with the in-house laboratory information system called the African Laboratory Information System (ALIS). Drug bug data sheets are generated from the national microbiology reference laboratory and are then shared with the AMR-NCC.

**Figure 4:**
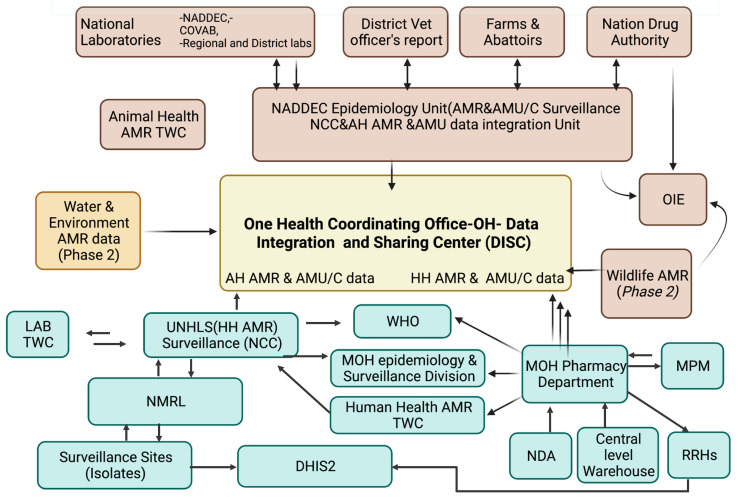
Illustration of the data sharing architecture with Human Health data linkages to One Health. This is colour coded according to the human health (green), animal health (brown), water and environment as a light brown shade.

The National AMR Coordination Centre hosts the database, and according to the established data-sharing guidelines, the sharing of these data will follow the Data Sharing and Information Centre (DISC) yet to be established under the One Health for AMR. Currently, the architectural design of the data-sharing centre exists. { TC “Figure 4: Components of the AMR surveillance system” \f C \l “1” } 

The National Health Laboratories and Diagnostic Services, together with the Fleming Fund country lead grantee, have embarked on an in-service microbiology training program for laboratory technologists in microbiology in both human and animal health. These efforts have resulted in developing a National Microbiology training curricula for in-service laboratory carders in both human and animal health. A draft training roadmap has been drawn up with a two-week facility-based mentorship plan to improve the microbiology bench skills of laboratorians in the fight against resistance.

#### 1.1.5. Surveillance for AMR Stewardship Optimal Use, Access and Consumption{ TC “Surveillance for AMR Stewardship Optimal Use, Access and Consumption” \f C \l “1” } 

As guided by the AMR-NAP, the development of a successful national AMU/C surveillance program is on-going currently with the process of policy and guideline development, and reviews as pre-requisite to ministry approval for operationalization. The protocols and guidelines are currently being drafted and presented to the ministries as well as the One Health technical working committees to guide and update the surveillance plans.

Antimicrobial use surveillance and monitoring are widely acknowledged as critical components of the response to antimicrobial resistance (AMR) and are one of the five strategic priorities of the National Action Plan (NAP) on AMR. To be most effective, antimicrobial surveillance systems should be coordinated and complementary to each other in respect of the National Action Plan. A cohesive surveillance system looks to the One Health approach to provide a more complete picture of AMR and AMU and facilitate analyses of trends over time and space and of relations among sectors. One of the major objectives of the Fleming Fund grant is to support the integration through increased collaboration between stakeholders to implement a One Health AMR surveillance programme. To this effect, country AMU/Stewardship indicators, guidelines and protocols for data collection and quality assurance have been developed. In addition to ongoing efforts to promote the inclusion of AMU surveillance data in the reporting at the Data information sharing centre, our preliminary results demonstrate the utility of this data integration.

At a sub-national level, there has been on-going work in the line of surveillance for stewardship in various selected hospitals under the partnership support of Tropical Health Education Trust (THET). THET is working with Commonwealth Pharmaceutical Association (CWPAMS) to implement activities in line with Uganda’s National AMR Action Plan 2018−2020 in six local hospitals in Uganda. These hospitals include Jinja, Fort Portal, Gulu, Kawempe Regional Referral Hospitals, Entebbe Grade B Hospital and St. Mary’s Hospital Lacor-Gulu. These hospitals are linked to partner hospitals in the United Kingdom to promote responsible access and use of antimicrobials as well as addressing the gaps in infection prevention and control (IPC) within the local facility. To do so, routine activities of the Medicines and Therapeutic Committees have been revamped, and Infection, Prevention and Control Committees have been constituted. All of this has been done with a strong focus on linking laboratories to clinical teams. For example, the Fort Portal Regional Referral Hospital laboratory team are now engaged in clinical decision-making with regard to antimicrobial agent prescriptions. These efforts are supported with capacity building of relevant stakeholders in collaboration with Salford University, Health Education Institution of the United Kingdom, and Pharmaceutical Society of Uganda (PSU). Taken together, these efforts will change attitudes on how prescription decisions are made and inherently contribute to prudent use of currently available antimicrobials.

#### 1.1.6. Approaches for AMR Data Management in Human Health{ TC “10.0 METHODS AND APPROACHES FOR AMR DATA MANAGEMENT IN HUMAN HEALTH” \f C \l “1” } 

AMR surveillance in low- and middle-income settings [10] reflects Uganda’s perspective. Raw data on ten national priority pathogens classified for country surveillance were collected following a recent study of acute febrile illness (AFI) alongside the clinical samples received by these laboratories for routine patient care [11]. As an initial step, Uganda designated sentinel sites to perform AFI surveillance studies with the support of the CDC and the Global Health Security Agenda. The project and its data helped inform the initiation of the national surveillance program to select sites with minimal capacity. It was this first set of data that was used in the first country AMR WHO-GLASS report. Isolated priority pathogens included *Acinetobacter* spp., *E. coli*, *K. pneumonia*, *N. gonorrhea*, *Salmonella* spp., *Shigella* spp., *S. aureus*, *S. pneumoniae*, *Pseudomonas* spp., and *H. influenza*.

**Figure 5:**
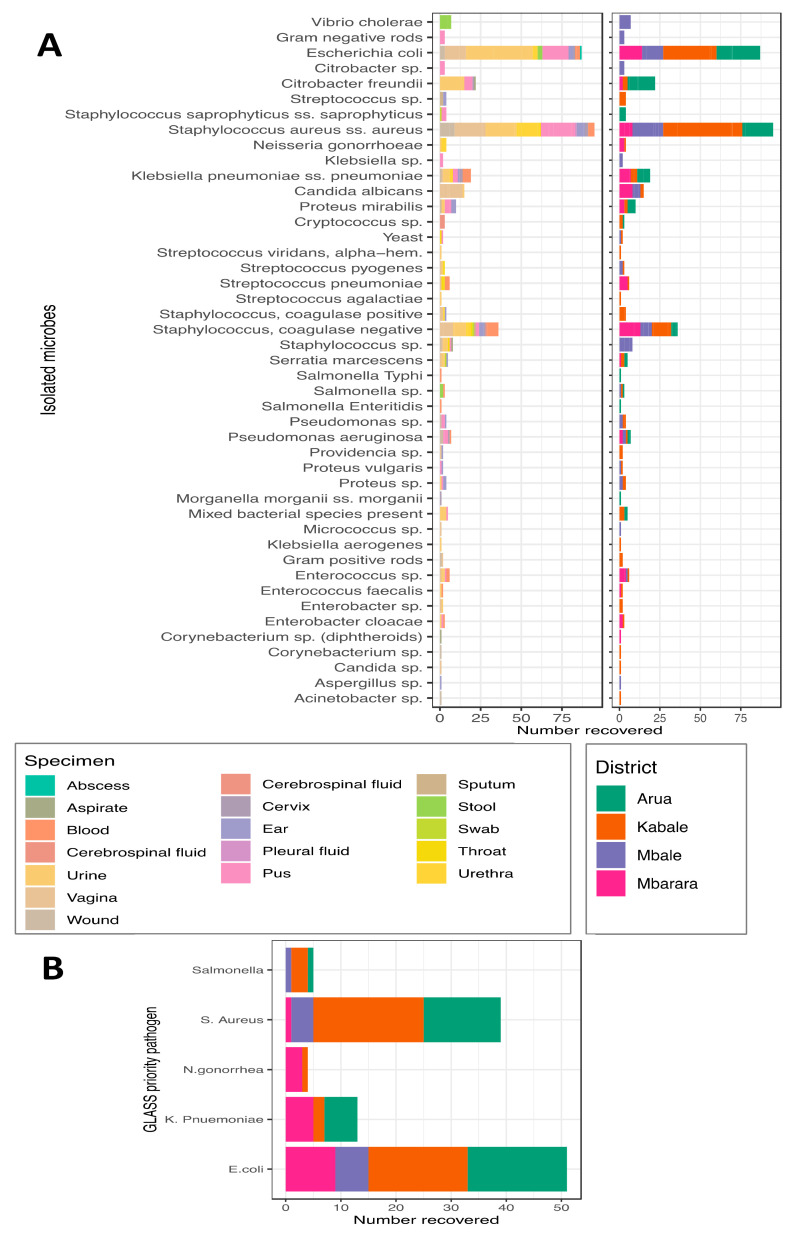
Microbes recovered from the cultured samples in Figure 3**.** Here, (**A**) has two panels, the first showing distribution by specimen and the second by district of origin. (**B**) shows the GLASS priority pathogens recovered from each of the district sentinel sites.

Following a routine case-based surveillance approach towards priority specimens (blood, urine, stool, genital swabs and CSF), these samples are sent to microbiology laboratories for the purposes of clinical testing at the four sentinel sites. Here, using patient-level data tools, epidemiological, clinical and demographic data were also collected alongside the microbiology data. After primary microbiology cultures and sensitivity testing, isolates are sent to the National Microbiology Reference Laboratory (NMRL) for validation and archiving. At the end of every quarter of a year, the NMRL must conduct site visits for retrospective on-site data cleaning and support supervision for improved microbiology culture and sensitivity laboratory procedures. It is noteworthy that for some sites, data cleaning and entry are performed in real-time using WHONET software (Figure 5A,B) displays the preliminary results and outcomes of these initiatives.1.1.7. Processing and Quality Control for AMR Data{ TC “Process Quality Control for AMR Data” \f C \l “1” }.

Sentinel sites are connected to a national microbiology network that includes the National Microbiology Reference Laboratory, academia, private sector and other research laboratories performing microbiology culture and sensitivity testing. All laboratories in the network are required to refer isolates to the National Microbiology Reference Laboratory for validation and archival in the biorepository. In addition, all regional reference laboratories participate in the Laboratory Quality Management Systems for internal quality control. A new scheme for microbiology in-country EQA was recently rolled out by the National Microbiology Reference Laboratory, beginning with Gram’s reaction and panel test to 16 laboratories in the network. Antimicrobial susceptibility testing is performed following the CLSI guidelines.

**Figure 6:**
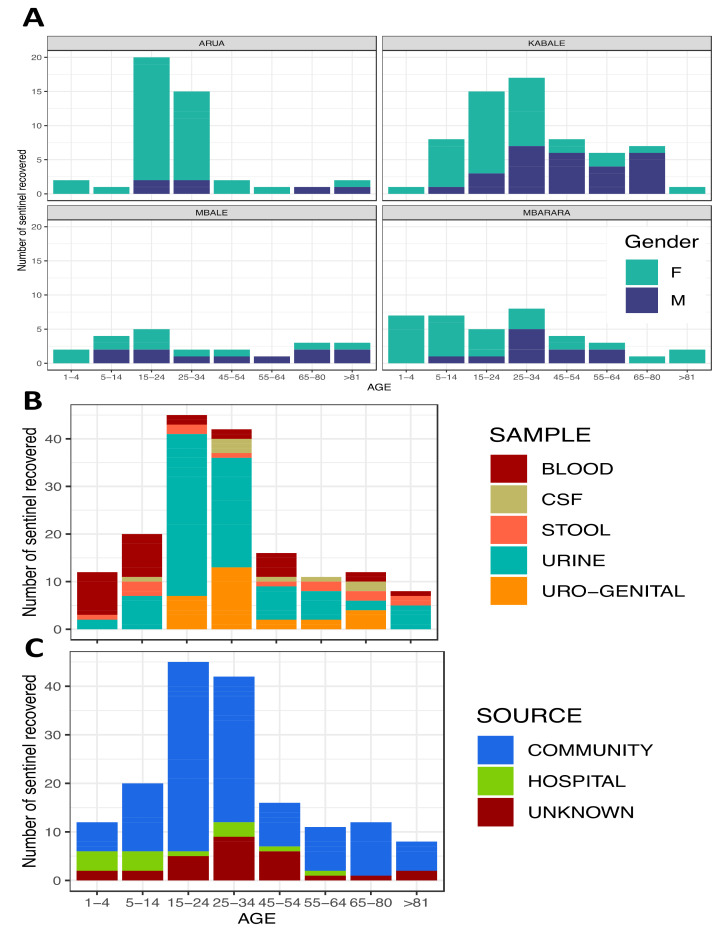
Preliminary analysis of microbes recovered from specific samples. (**A**) shows the distribution of these organisms by district, age group and gender. (**B**,**C**) show the same distribution by specimen collected and source, i.e., community or hospital.

The WHONET is integrated with the existing laboratory information systems as customised by the AMR surveillance in Uganda; these include the African Laboratory Information System (ALIS). Figure 6A–C displays the functioning units of the AMR surveillance system and how the data generated displays the WHO-GLASS priority pathogens and specimens. This is ideal for any AMR surveillance system that focuses on AMR priority pathogens and specimens. When integrated, these two software platforms will provide the basic digital infrastructure for local surveillance and reporting to WHO-GLASS and the AMR- National Coordination Centre of the Ministry of Health. The exported data sets were preliminary analysed and generated preliminary reports as presented in the results section below.

### 1.2. Preliminary Results and Outputs

#### Preliminary Antibiotic Resistance Profiles

**Figure 7:**
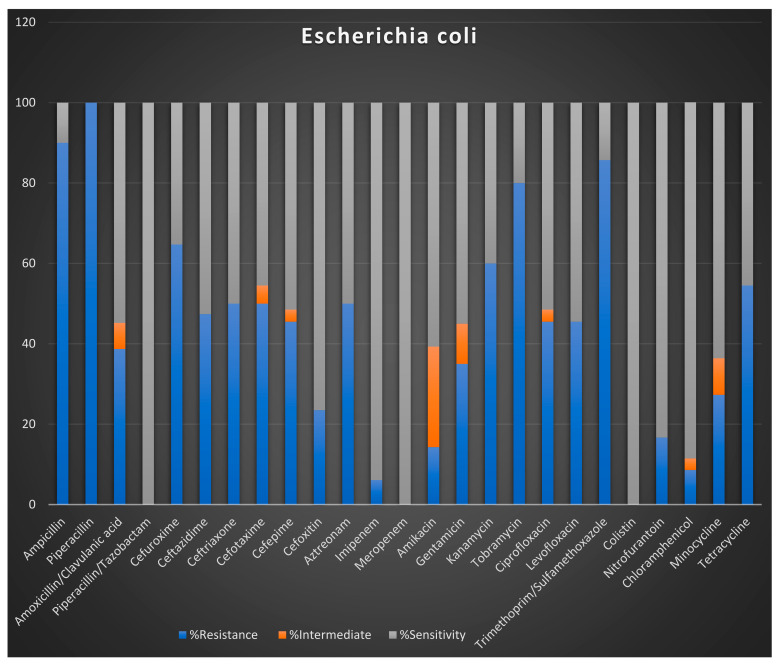
Stacked column graph for *E.* *coli* is for Arua Health Region isolates for AST patterns validated by the National Microbiology Ref. Lab for 2019. Data source and analysis by NMRL-AMR NCC-Biostatician.

**Figure 8:**
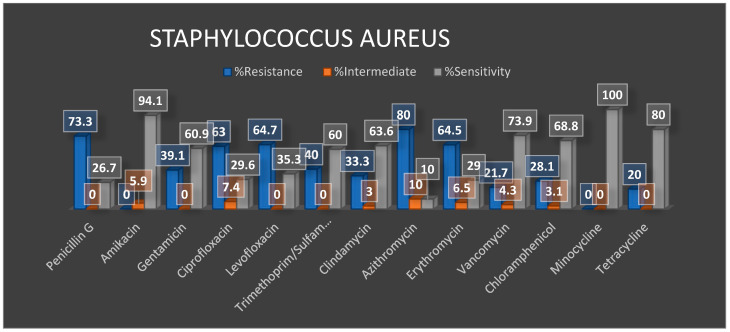
Distribution of antimicrobial susceptibility profiles of *S. aureus* across different available antibiotics.

This analysis focused on two susceptible GLASS priority organisms, i.e., *E. coli* and *S. aureus*. These were tested on 25 commonly used antibiotics, as shown in the stacked Bar graph in Figure 7. We noted a high prevalence of resistance of *E.coli* to ampicillin and piperacillin, exhibiting over 95% resistance. Although this remains to be validated, the subsequent analyses denominator number of isolates still suggest that these two antibiotics have almost exhausted their utility for this microbe in this population, thus an impending gross resistance of bacteria to the molecules. Furthermore, over 60% of the *E. coli* were resistant to cefuroxime, tobramycin and trimethoprim/sulfamethoxazole as depicted first in recent studies. Figure 8 further details the antimicrobial susceptibility profiles of *S. aureus* given the listed antibiotic discs used in the laboratory. Picking out *S. aureus* highly underscores its resistance profiles to give a high light for Methicillin-Resistant Staphylococcus Aureus (MRSA) as it will be highlighted in our future situation report release.

Due to their use for prophylaxis against other infectious diseases in immunocom-promised patients [12], trimethoprim/sulfamethoxazole are examples of antibiotics that have been overused, here we relatively draw our assumptions and infer that the over use in prophylactic preventions exposes the bacteria to high microbial resistance as well as mounting endemic resistance to the cephalosporins class, leading to further overuse by clinicians hence a broad resistance.

The antibiotics with the least resistance include piperacillin-tazobactam, meropenem, imipenem and colistin; indeed, the data on usage and consumption below shows direct relationships with these patterns. On the other hand, 80% of *S. aureus* isolates were resistant to azithromycin. Many colistins and meropenem are antimicrobials used in single and combination therapy for colistin-resistant bugs [13]. However, in Ugandan public hospital settings, they are indicated as reserve drugs in the public sector and can only be dispensed with a physician’s approval. Therefore, the limited resistance observed could be linked to the limited use component because their availability for consumption is limited due to high cost and reserve use in hospital settings [14]. Other antibiotics to which *S. aureus* showed higher levels of resistance included penicillin, Ciprofloxacin and levofloxacin. The least resistant were amikacin and minocycline.

**Figure 9:**
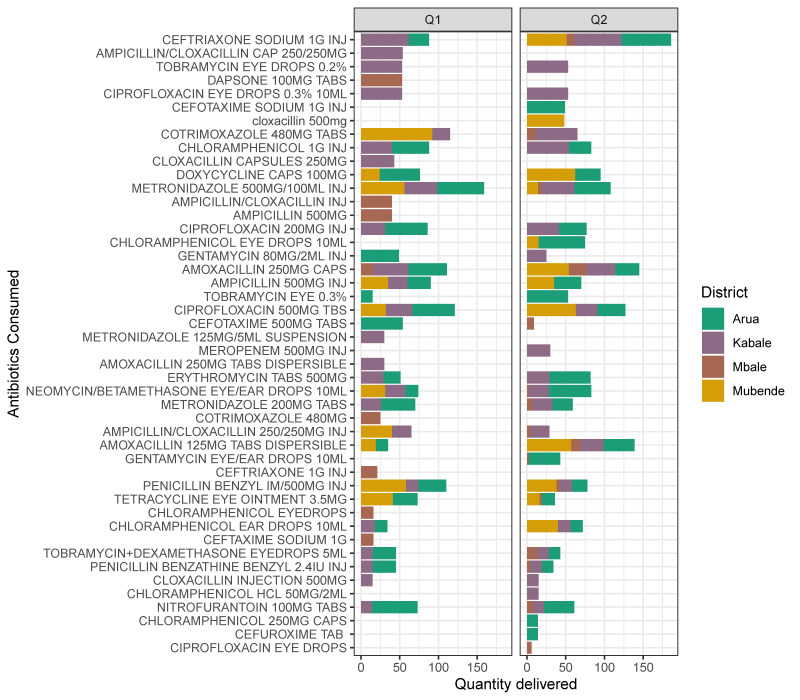
Antibiotic consumption performance among four Sentinel Sites, Data Collection by AMU/C Technical Working Group with support from IDI-GHSA Project.

From Figure 9, the integration of AMR and AMU/C data means examining the relationship between these two parameters across temporal and spatial scales. Here, the integrated data were limited to four districts Arua, Kabale, Mbale, Mbarara. Here we show the volumes and diversity of antibiotics delivered for use at each of the referral hospitals. Indeed, the most consumed antibiotics, i.e. Cefriaxone, Cotrimoxazole, Amoxaxcilin, Ciprofloxacin, also have the highest resistance (Figure 7 and Figure 8). We also examine Arua referral hospital as the exemplar for the sentinel sites to examine how drugs are used in the out-patient department(OPD). Here we also note that Ciprofloxacin is among the most prescribed antibiotics. Metronidazole, a duo purpose, i.e. antibiotic and antiprotozoal, is the most prescribed. This provides an initial overview of how prescription drives the trends of AMR observed.

## 2. Discussion

This situation analysis report focuses on the human health surveillance framework design for AMR as presented in the figures and preliminary data analysis above. It provided preliminary outputs from this system that the implementer can then use to examine Uganda’s AMR-NAP’s performance and inform plans of scaling up the AMR surveillance network from four sentinel sites to cover all the referral hospitals. It further demonstrated with evidence the practical integration and communication between components of the surveillance system as it was mapped for data collection, quality and quantity, and microbiological recovery in laboratories and the diversity of pathogens detected to examine the performance and utility of this sentinel surveillance system.

The evidence illustrates that structural designs for data collection, processing, quality assurance and sharing through digital surveillance infrastructure systems and linkages are ideal and worth benchmarking in low and limited settings as approaches to AMR surveillance using existing systems versus investments in operations and the implementation of scientific research.

It is noteworthy that most of the microbes recovered here originated from clinical samples with no clear cut-off point to determine community- and hospital-associated infections. This is still a challenge in the clinical management of infectious diseases, as most clinical settings have not clearly defined the criteria for the classification of these infections. Similarly, there was a significant increase in the prevalence of *E. coli* and *K. pneumoniae*, producing CTX-M-type extended-spectrum beta-lactamase and car-bapenemase in patients from a rural community with community-acquired infections; these infections were then transmitted to hospital communities, significantly contributing to hospital-associated infections [15]. This makes infection prevention and control strategies in each of these sentinel sites ideal for addressing the transmission of resistant bugs. The data discussed included a small but significant number of samples whose origin was not captured. Therefore some epidemiological utility was inherently lost.

The results show between 2019 and 2020, 1209 samples were received from four of the 12 sentinel sites across the country for culture and identification of organisms, as shown in Figure 3, Figure 5, Figure 6 and Figure 8. The distribution of the cultured samples is presented in Figure 3; these show a normal distribution by age with modal age at 15−24, suggesting that the majority of samples were collected from patients within this age group. Most of these samples originated from the Arua district, whereas the Kabale district accounted for the smallest number. This preliminary output should be viewed as an early indication that the surveillance structure are functional. The extent to which all structures function will be assessed as more data is collected and as more sentinel sites are added. Nonetheless, the preliminary data reveals differences in culture and sensitivity done versus the samples collected, i.e. Kabale district.

### 2.1. Antibiotic Consumption and Use among the Four Sentinel Sites That Are Regional Regional Referral Hospitals

It is ideal that for any functional AMR surveillance system, antimicrobial use and consumption data be analysed alongside the antimicrobial resistance data for resistant organisms. This is ideal to understand how the use of antibiotics, either optimally or sub-optimally, can affect the development of resistance among the organisms [16], provides evidence that reducing the consumption of antibiotics would be enough to slow down the dissemination of resistance in the downstream environment, reducing the exposure of microbes to antimicrobial agents. Therefore, parallel analysis of AMR and AMU/C provides evidence of how and why irrational use and consumption is the hallmark driver of re-emerging resistance.

**Figure 10:**
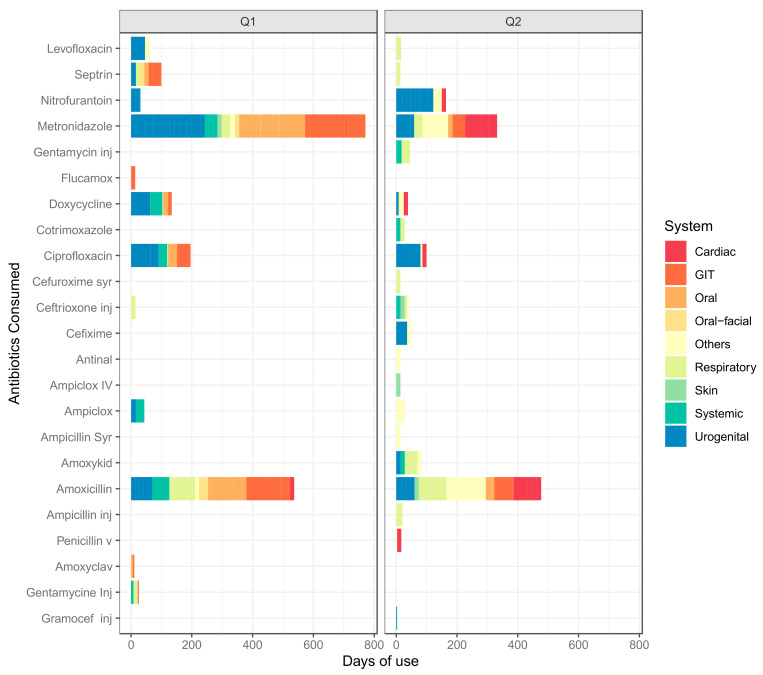
Antibiotic prescription at the outpatient department of Arua regional referral hospital for Quarter 1& Quarter 2 of 2019. The figure is a bar plot whose axes were flipped to improve visualisation. The *y*-axis shows the cumulative number of days the patients at this department were on any given antibiotics while the *x*-axis shows the type of antibiotics. The color fill shows the system to which the disease under management clusters. For example, a Urinary tract infections would cluster under Urogenital system. Note that majority of the “others” are malaria cases.

In general, Figure 9 and Figure 10 above provides preliminary insights and justification for an integrated AMR and AMU/C data analysis. For example, although the data in Figure 9 and Figure 10 is limited to the four site’s consumption of drugs supplied by the national medical stores, we note that AMU/C is associated with AMR; above all, the use of “access” drugs is higher than “watch” drugs in both districts.

Figure 9 suggests that the consumption and use of antimicrobials might be driven by availability, access and affordability of antimicrobials and not necessarily efficacious/suitability. Furthermore, in Figure 10, the pathological conditions were the most recorded probably highlight the preoccupation of the age range shown in Figure 3.

The preliminary results also suggest that antibiotic consumption is higher for certain antibiotic molecules i.e. cephalosporins and Ciprofloxacin. Similarly, albeit smaller, another peak is observed between April and June. The most consumed antibiotics include amoxicillin, ceftriaxone, cefuroxime, ciprofloxacin tablets and metronidazole However, the current data analysed was insufficient to reveal the bottlenecks’ gist in regards to using and consumption drivers and other compounding factors. Still, this report further sets the precedence by indicating the efforts invested in understanding the core of AMR surveillance with drug-bug resistance patterns compared with the use and consumption component.

Therefore, the role of public awareness, education and training is ideal for addressing AMR across all key stakeholders. This could scale from the public and community levels to policymakers as well as health workers’ groups. The Uganda National Action Plan (on http://www.cphl.go.ug/policy-documents (accessed on 18 August 2021)) powerfully underscores the role of public AMR awareness, education and training. This is a strategic objective in the NAP, with key specific outputs incorporating AMR into health worker training curricula for both pre-and post-service healthcare workers, with didactic and hands-on practicum training for post-in-service healthcare workers.

Similarly, another study [17], explored the use of an AMR dictionary as an educational tool to improve public dialogue campaigns in the form of public drives and dialogue in both lower and secondary schools as didactic training with the involvement of policy and civil society workers.

Finally, it is imperative that health practitioners’ knowledge, Attitudes and Practices (KAP) towards addressing all the facets and drivers of AMR be emphasised and incorporated into the pre-service and post-service curriculum as this is ideal for changing attitudes and promoting awareness among the medical fraternity. A health communication strategy with the perspective of AMR in One Health could be ideal for combating AMR across all sectors. 

### 2.2. Current Limitations of the Surveillance System{ TC “11.0 CURRENT LIMITATIONS OF THE SURVEILLANCE SYSTEM” \f C \l “1” }

The fundamental limitations of the system are as follows:

- Non-uniformity of testing isolates against different priority organisms, thus a need to adopt the standardized national microbiology protocols.
- Lack of awareness leading to less utilization of the microbiology laboratories, hence less demand.
- Inadequate microbiology supplies and logistics. This has failed to provide results consistent with culture and sensitivity test requirements. A large share of these supplies is donor-funded, thus raising questions for the program’s sustainability at the national level.

Lack of human resource capacity for microbiology susceptibility testing.

The small number of sentinel sites submitting data limits the magnitude of the data. Significant efforts are needed to scale up to other sites in the shortest time possible.

## 3. Conclusions and Lessons Learned { TC “12.0 CONCLUSION” \f C \l “1” }

Uganda is making strides and progress concerning developing and implementing a functional AMR surveillance strategy for human health. Although numerous challenges still exist, following the laboratory health system strengthening approach, the fundamental issues are the health infrastructure, its integration, capacity building and operation.

Preliminary results suggest high resistance to common antibiotics that maps to usage and consumption across spatial scales. We emphasize that these results are preliminary and meant to set a stage for continued implementation of Uganda’s plan as a fulcrum of support for the developing system.

## Data Availability

The data used to generate these graphs can be obtained and sourced from the National database. For privacy, these data are anonymized in the form of isolates and AST formats. This database has been built up to form the national AMR Database for AMR surveillance, from which we can retrieve AMR-WHO-GLASS annual reports.

## Supplementary Materials

The following are available online at https://www.mdpi.com/article/10.3390/tropicalmed6040178/s1, Figure S1: How samples and data are captured from the surveillance sites, Table S1: National Microbiology Laboratory Request and Results Reporting Form, Table S2: National AMR Surveillance Indicators.

## Abbreviations

NDA	National Drug Authority
RRHs	Regional Referral Hospitals,
AMR TWC	Antimicrobial Resistance Technical Working Committee
HH	Human Health
DHIS	District Health Information System
AMUC	Antimicrobial Use and Consumption
NCC	National Coordination Centre
NADDEC	National Animal Disease and Diagnostic Centre
AH	Animal Health
CLSI	Clinical Laboratory Standards Institute
OIE	World Organisation for Animal Health
COVAB	College of Veterinary and Biosecurity
DVO	District Veterinary Officer
OH	One Health
ESD	Epidemiological Surveillance Division
MOH	Ministry of Health
HMIS	Health Management Information System
DHIS2	District Health Information System 2
NFP/NCC	National Focal Point/National Coordination Centre
ALIS	African Laboratory Information System
BBSRC	Biological Biotechnology Research Council
