# Supplementary material for: Antimicrobial Resistance Situational Analysis 2019–2020: Design and Performance for Human Health Surveillance in Uganda
Source: Trop Med Infect Dis. 2021 Sep 29;6(4):178. doi: 10.3390/tropicalmed6040178 (PMC8544686; doi:10.3390/tropicalmed6040178)
Supplement: Supplementary file 1 [file tropicalmed-06-00178-s001.zip › tropicalmed-1365809-supplementary.pdf]

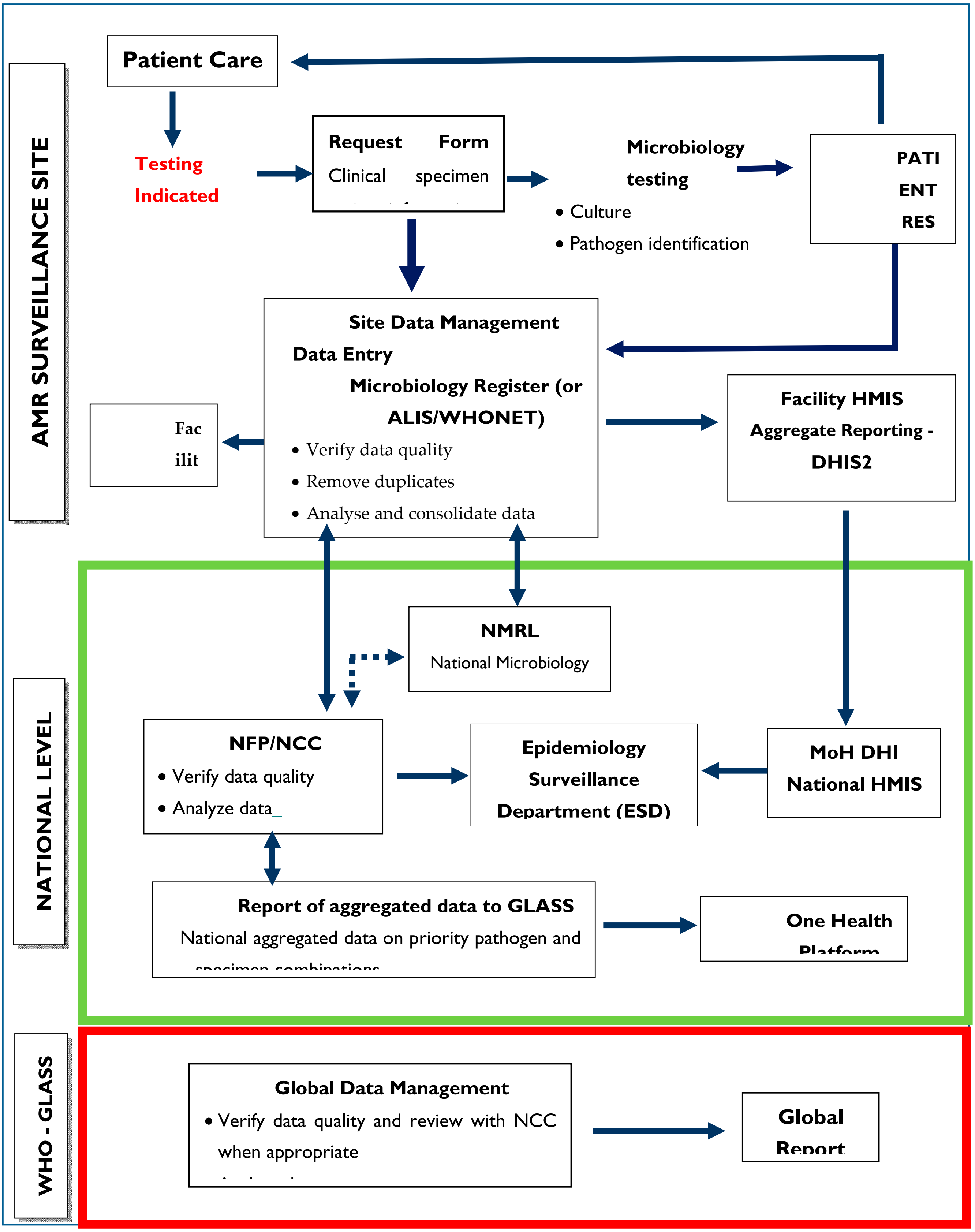

AMR SURVEILLANCE SITE
Patient Care
Testing Indicated
Request Form
Clinical specimen
Microbiology testing
• Culture
• Pathogen identification
PATI ENT RES
Site Data Management
Data Entry
Microbiology Register (or ALIS/WHONET)
• Verify data quality
• Remove duplicates
• Analyse and consolidate data
Fac ilit
Facility HMIS
Aggregate Reporting -
DHIS2
NATIONAL LEVEL
NMRL
National Microbiology
NFP/NCC
• Verify data quality
• Analyze data
Epidemiology Surveillance Department (ESD)
MoH DHI
National HMIS
Report of aggregated data to GLASS
National aggregated data on priority pathogen and
One Health
WHO - GLASS
Global Data Management
• Verify data quality and review with NCC when appropriate
Global

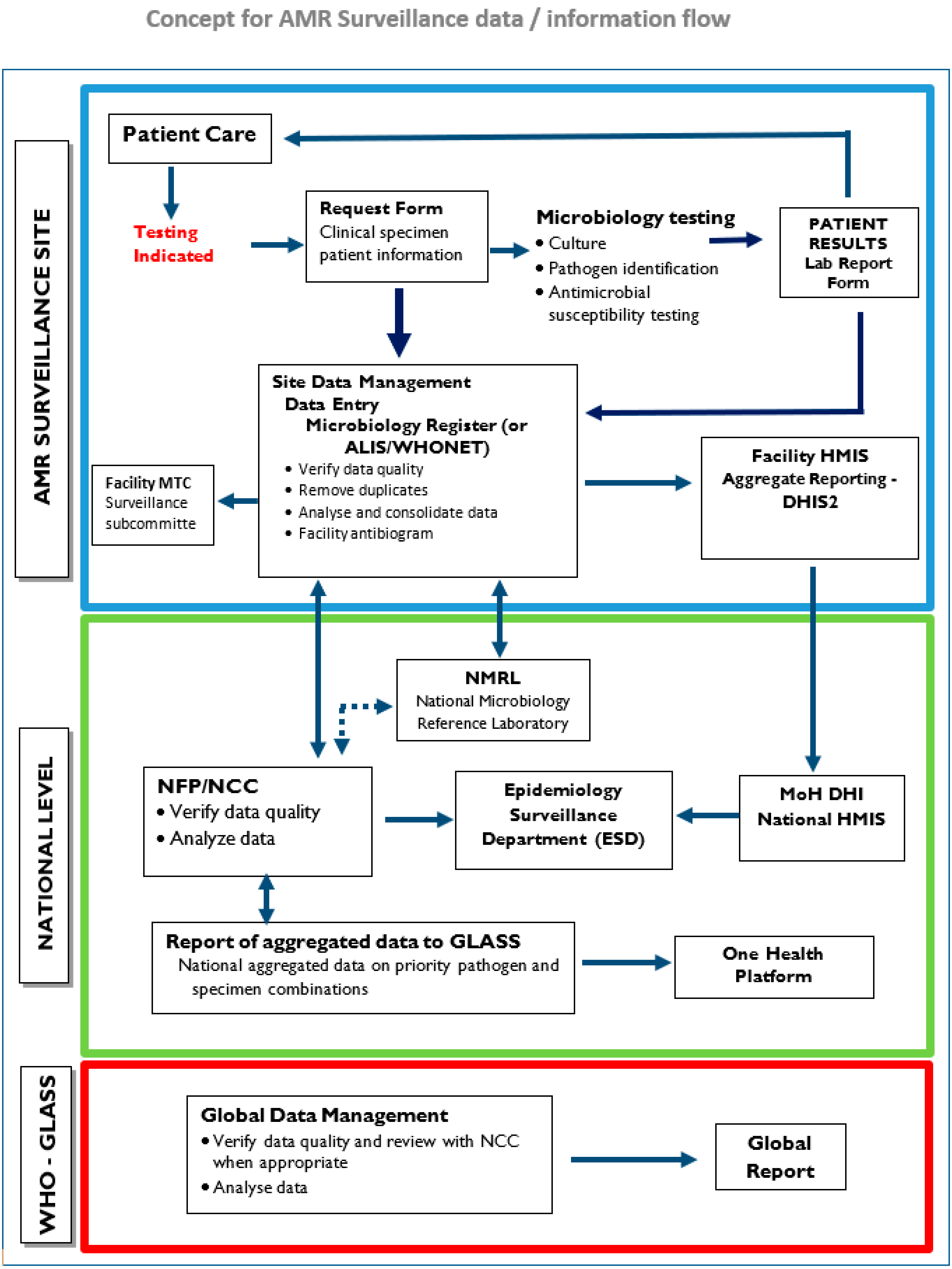

Figure S1. How samples and data are captured from the surveillance sites.

Table S1. National Microbiology Laboratory Request and Results Reporting Form.

National Microbiology Laboratory Request and Results Reporting Form

THE REPUBLIC OF UGANDA

MINISTRY OF HEALTH

NATIONAL MICROBIOLOGY LABORATORY REQUEST AND REPORT FORM

Form Serial Number

1. Facility Information

Facility Code:<<*auto selection of facility code from a drop down menu of facility codes from master facility list*>>		Facility Name:<<*auto selection of facility name from a drop down menu of facility names from master facility list*>>
Facility Ownership:<<*auto selection of facility ownership from a drop down menu comprising: Public, PHP, PNFP, Other (Specify)*>>		Facility Level:<<*auto selection of facility level from a drop down menu comprising: Public: NRHL, RRHL, GHL, HC4L, HC3L*>>, << *Private: Level 3, Level 2, Level 1*>>
District Code:<<*auto selection of district code from a drop down menu of district codes from MOLG district list*>>	District Name:<<*auto selection of district name from drop down menu of district names from MOLG district list*>>	Health Region:<<*auto selection from a drop down menu comprising the list of health regions*>>

2. Information on Requesting Officer

Surname:	Other names:	Cadre: <<*drop down menu containing: medical officer, clinical officer, nurse, public health officer, Other (specify) …..*>>	Signature:
Telephone:		Email address:	
Date of request: *DD/MM/YY*		Time of request: *HH:MM*	

3. Patient Information

Patient National Identification Number (NIN):	OPD/Health Facility Number*(for patients without a NIN, or facilities that do not use NIN as patient identifier)*:	Unique Laboratory Identification Number (ULIN):<<*field format: facility code-patient initials (4 letters max)-lab auto generated number*>>

Surname:	Other names:	Date of Birth: *DD/MM/YY*	Age: <<*age calculator: for only patient who don't know date of birth that automatically calculates it*>>
Sex:<<*Male, Female*>>	Occupation:	Telephone Number(s):	Email address:

**Residence details**			**Work place details**		
District:<<*drop down menu comprising: list of districts*>>	Sub county:<<*drop down menu comprising: list of sub*	Village: <<*drop down menu comprising: list of villages within a*	District:<<*drop down menu comprising: list of districts*>>	Sub county:<<*drop down menu comprising: list of sub counties within a*	Village:<<*drop down menu comprising: list of districts*>>

	counties within a specified district>>	*specified sub county>>*		*specified district>>*	

Surname of Patient's Next of Kin:	Other names:	Telephone Number(s) of Patient's Next of Kin:
Name of Ward/Specialized Clinic:		

4. Clinical Information*(tick where applicable)*

Major Clinical Signs and Symptoms	☐ Fever<br>☐ Vomiting<br>☐ Nausea<br>☐ Diarrhoea	☐ Abdominal pain<br>☐ Headache<br>☐ Loss of appetite<br>☐ Conjunctivitis	☐ Sore throat<br>☐ Rash<br>☐ Constipation<br>☐ Night Sweats	☐ Persistent Cough<br>☐ Bloody Stools<br>☐ Enlargement of glands<br>☐ Bleeding through Body Openings	☐ Other symptoms (specify)

Hospitalized for more than 2 calendar days? Y/N			
Patient transferred from another health facility? Y/N	*If YES, Name facility that has transferred patient:*		
Has the patient been on antibiotics during current infection?	☐ YES	If YES, specify drugs administered to patient :<< *drop down with: antimicrobials administered, Other (specify) ………>>*	Duration on drugs: ………days
	☐ NO		

**Provisional diagnosis/disease suspected *(tick all options that apply)***				
**4.1. Epidemic prone diseases**				
☐ Cholera<br>☐ Anthrax<br>☐ Measles	☐ SARI<br>☐ Yellow fever<br>☐ Typhoid fever	☐ Brucella<br>☐ Dengue<br>☐ Plague	☐ Diarrhoea with blood (Shigella Dysentery)<br>☐ Chikungunya Meningococcal meningitis	☐ Acute Haemorrhagic Fever syndrome (Ebola, Marburg, Rift Valley, Lassa, Crimean Congo, West Nile Fever)

**4.2. Diseases targeted for eradication or elimination**		**4.3. Diseases or events of international concern**	
☐ Buruli ulcer<br>☐ Neonatal tetanus<br>☐ Leprosy<br>☐ Noma	☐ Onchocerciasis<br>☐ Dracunculiasis<br>☐ Lymphatic filariasis<br>☐ Poliomyelitis	☐ Smallpox<br>☐ SARS<br>☐ Human influenza due to a new subtype	☐ Other public health event of inter (national) concern (infectious, zoonotic, food borne, chemical, radio nuclear, or due to unknown condition)

**4.4. Other major diseases, events or conditions of public health importance**		
☐ Trypanosomiasis<br>☐ Diabetes mellitus<br>☐ Rabies<br>☐ Malaria<br>☐ Maternal	☐ Trachoma<br>☐ STIs<br>☐ Tuberculosis<br>☐ Acute viral hepatitis<br>☐ Mental health (Epilepsy)<br>☐ Injuries (Road traffic Accidents)	☐ HIV/AIDS (new cases)<br>☐ Severe pneumonia less than 5 years of age<br>☐ Malnutrition in children under 5 years of age<br>☐ Adverse events following immunization (AEFI)

deaths<br>☐Hypertension		☐Diarrhoea with dehydration less than 5 years of age<br>☐Others (specify)

5. Specimen Information

Tick the type of specimen					
☐Sputum<br>Vomitus<br>☐Urine<br>☐<br>☐CSF<br>☐Stool<br>☐Wound<br>☐<br>Beverage	☐Food remains<br>☐Water<br>☐Eye swab<br>Vaginal<br>Aspirate<br>☐Se-<br>☐rum/plasma<br>☐Blood culture	☐Gastric<br>☐Scalp<br>☐Biopsy/tissue<br>Skin snip/nail/hair<br>Abscess/pus<br>☐<br>☐swab<br>☐Sinus<br>☐Bone marrow	☐Nasal washing<br>☐Rectal<br>☐Urethral<br>☐Nasopharynx (NP)<br>☐Bronchial brush<br>☐Throat<br>☐Dialysate	☐Vitreous<br>☐Bronchial wash<br>☐<br>☐<br>Catheter Urine<br>Environmental sample<br>☐Veterinary Specimen<br>☐Urethral Secretion<br>☐Cervical Secretion	☐Synovial Fluid<br>☐Bronchoalveolar lavage<br>☐Mucocutaneous<br>☐Urine – Suprapubic<br>☐Transbronchial bi-<br>☐opsy<br>Other (specify)

Date of specimen collection: *DD/MM/YY*	Time of specimen collection: *HH:MM*	Date of specimen dispatch to testing lab: *DD/MM/YY*	Time of specimen dispatch to testing lab: *HH:MM*
Surname of officer receiving specimen in testing lab:	Other names of officer receiving specimen in testing lab:	Date when specimen is received in testing lab: *DD/MM/YY*	Time when specimen is received in testing lab: *HH:MM*

5.1. Type of Test Requested

**Examination required:**			
Indicate the requested test from the type of specimen.			
Specimen	Test request	Specimen	Test Request

6. Receiving specified lab

**Details of receiving officer at reference/specialized lab**		
Surname:	Other names:	Cadre:
Telephone:		Email address:
Date of receiving: DD/MM/YY	Time of receiving: HH:MM	

7. Specimen Quality (to be filled by ONLY LABORATORY PERSONNEL at receiving laboratory)

If specimen is rejected, specify reason for rejection	☐ Specimen without lab request form<br>☐No sample label or identifier<br>☐ Unclear sample label	☐ No test ordered on lab request form of sample<br>☐Wrong sample label<br>☐ Sample type unacceptable for required test	☐ Other (specify)

If specimen is accepted, comment on quality of specimen*(tick*	☐ Poor<br>☐ Fair	☐ Inadequate specimen volume<br>☐☐ Inappropriate type of sample container<br>☐ Inappropriate transport media of sample<br>☐ Poor shipment condition *(e.g., lack of cold*	Delayed sample delivery time/too old sample<br>☐Date of sample collection not specified<br>☐ Time of sample collection not specified<br>☐

where applicable)		*storage)*	☐ Other (specify)
	☐ Good		

National AMR Surveillance indicators based on the national needs, both institutional and interventional. Among the institutional, these include the number of sites to perform microbiology C/S and the number of sites/lab reporting. The interventional needs refer to the resistance patterns and which organisms present with emerging resistance.

Interventional indicators to be reported on monthly: the volume of work being performed; what are the prevailing organisms; what are their sensitivity patterns and which organisms show emerging resistance

Highlighted major resistances include: prevalence of MRSA; 3rd generation cephalosporin resistance; carbapenem-resistant Enterobacteriaceae; vancomycin-resistant Enterococcus and colistin resistance.

Institutional Indicators (to be reported quarterly include: the number of sites performing culture and sensitivity testing, the sites are reporting their culture and sensitivity findings to NHLDS.

Specimen volume indicators include:

Table S2. National AMR Surveillance Indicators.

SPECIMEN	TOTAL NO RECEIVED	NO CULTURED	NO REJECTED (%)	SIGNIFICANT GROWTH (%)	CONTAMINATION (%)
Blood					
Urine					
Stool					
CSF					
Urethral swabs					
Cervical swabs					
Aspirates					
Pus swabs					
Other swabs					
Sputum					
Isolates					

PATIENTS DEMOGRAPHICS

	FEMALE	MALE
< 1 month		
1 – 11 months		
12 – 59 months		
60 months – 11 years		
12 - 18 years		
> 18 – 30 years		
31 – 45 years		
45 – 65 years		
> 65 years		

HOSPITAL ACQUIRED AND COMMUNITY ACQUIRED INFECTIONS

	% No of patients
HOSPITAL ACQUIRED INFECTIONS	
COMMUNITY ACQUIRED INFECTIONS	
AMR Related Mortalities	

8. Microbiology Laboratory Results/Report Section

8.1. Laboratory Findings

Tests	Results	Tests	Results
Appearance		Gram stain	
Bilirubin		ZN stain	
Glucose		Wet prep	
Ketones		Modified ZN	
Lymphocytes		Differential	
Nitrite		Crag	
PH		Neutrophils	
Protein		RBC count	
Red blood cells		Total protein	
Specific gravity		India ink	
Urobilinogen		WBC count	
Leukocytes		Cell Differential	
KOH		Reducing sugars	
Microscopy		Quantitative culture	
MRSA screening		Serum Crag	
HIV Serology		Total cell count	

Culture and Sensitivity Results:

#	Antibiotic type*(to be linked to the type of Microorganism)*	Organism 1 *(indicate organism name here)*		Organism 2 *(indicate organ-ism name here)*		Organism 3 *(indicate organism name here)*	
1							
2							
3							
4							
5							
6							

7							
8							
9							
10							
11							
12							
13							
14							
15							
16							
17							
18							
19							
20							
21							

Results Guide S – Sensitive R – Resistant I – Intermediate

**12. Test/Analysis Performed by:**			
Surname Name of officer:	Other Names of Officer:	Signature of officer:	Result dispatching date: *DD/MM/YY*
**13. Review Comment (s) on findings:**			
**Reviewed by:**			
